# Inguinal Lymph Node Dissection for Advanced Stages of Plantar Melanoma in a Low-Income Country

**DOI:** 10.1155/2020/8854460

**Published:** 2020-12-11

**Authors:** Ollo Roland Somé, Malick Diallo, Damien Konkobo, Nassirou Yabré, Valentin Konségré, Issouf Konaté, Sidy Ka

**Affiliations:** ^1^General Surgery Department, CHU Sourô Sanou (Burkina Faso), Bobo-Dioulasso, Burkina Faso; ^2^Orthopedic Surgery and Traumatology Department, CHU Sourô Sanou (Burkina Faso), Bobo-Dioulasso, Burkina Faso; ^3^Jolliot Curie Cancer Institute, CHU Dantec (Sénégal), Dakar, Senegal; ^4^Biology Department, CHU Sourô Sanou (Burkina Faso), Bobo-Dioulasso, Burkina Faso; ^5^Dermatology Department, CHU Sourô Sanou of Bobo-Dioulasso (Burkina Faso), Bobo-Dioulasso, Burkina Faso

## Abstract

**Background:**

Advanced stages of plantar acral lentiginous melanoma are common in Africa. Inguinal lymph node dissection (ILND) in these cases plays a critical role in disease-free and overall survival. Our study aims to share our experience in ILND for advanced plantar melanomas. *Methods and Study Design*. Four-year prospective study. *Patients*. We included all documented cases of advanced stage plantar melanoma with clinically detectable inguinal lymph node metastasis. Twenty-two of 27 patients identified—with mean age 56 years—underwent ILND. *Studied Variables.* Tumor patterns and stage, surgery, morbidity, oncologic pathology, and evolution were studied. Statistical software assessed the overall survival (OS).

**Results:**

Plantar lesions were all excised with a cancer-free margin (3 cm). ILND was performed for 22 patients with visible (*n* = 11), palpable (*n* = 7), and ulcerous (*n* = 4) lymphadenopathies. It was performed through an S-shaped (*n* = 11) or ellipse-shaped skin incision (*n* = 11). The tumors were AJCC stage III (*n* = 18) and IV (*n* = 2). We found high Breslow index tumor thickness (>3 mm) and an advanced Clark IV stage (*n* = 20). All operative wounds healed within 46 days (21–90). Wound healing was delayed by suture failure (*n* = 16), lymphorrhoea (*n* = 22), and infection (*n* = 18). After 29 months, three patients had complete remissions, seven had recurrences, and twelve patients had died. The overall survival (OS) at one year was 56%. In two patients with AJCC stage III disease, the OS was better (22 months).

**Conclusion:**

In low-income countries, ILND in advanced stages of plantar foot melanoma is a valuable surgical treatment option. Alongside ILND adjuvants, treatment must be available and accessible to improve survival.

## 1. Background

Melanoma is a malignant tumor from melanocytes rarely found in dark-skinned African or African-Americans [1–3]. In Europe, extensive nodular melanoma (NM) and superficial spreading melanoma (SSM) are common. The lentigo maligna melanoma (LM) is especially common on the face [4]. In Africa, plantar acral lentiginous melanoma is the most common form [2, 3, 5, 6]. Melanoma has high metastatic potential [1, 7]. According to the SEER 2019 database, the 5-year relative survival rate is 64% for melanoma with locoregional metastasis and 23% for those with distant metastasis [8]. Surgical management includes wide resection with inguinal lymph node dissection (ILND) in the case of lymph nodes metastasis [4, 7]. In low-income African countries, patients with tumors often cannot access timely specialized care [3, 9]. Therefore, presentation with AJCC stages III and IV is common [3]. Moreover, visible lymphadenopathies can progress to necrotic ulcers. Unmanaged melanomas severely impact patients' quality of life. In low-income countries with no access to radiotherapy and immunotherapy, surgery can be the only treatment option. Surgical management by large resection and ILND can improve the quality of life and overall survival (OS).

## 2. Methods

### 2.1. The Aim of the Study

The objective of our study was to share our experience of associated ILND for patients with advanced plantar melanoma managed at a tertiary care hospital in a low-income country.

### 2.2. Study Design

A 48-month prospective study from June 2015 to June 2019 was performed in our surgical department. It is the only specialized facility offering anatomopathological and surgical management for the western part of our country and other neighboring countries in the region (about seven million inhabitants).

### 2.3. Patients

Plantar melanoma (PM) diagnosis was made clinically. We included all patients with plantar melanoma (PM) with clinically detectable inguinal lymph node metastases (WHO grades 0, 1, and 2) [10]. Five patients were excluded due to low-grade tumors, AJCC grade I after excisional biopsy and staging (pT1a, N0, M0 and pT1b, N0, M0) (*n* = 2), and unresectable fixed inguinal lymph node metastasis (*n* = 3). CT scan (when available) and X-ray plus ultrasonography (US) were used to assess tumor extension. Despite metastatic evolution, ILND was performed in good clinical condition patients.

### 2.4. Studied Variables

We assessed tumor characteristics (Breslow index, mitosis, vascular invasion, etc.) and stage [11], surgical technique, surgical complications (neurovascular injury, wound dehiscence, healing time, and surgical site infection), oncologic pathology (resection margin and the ratio between invaded nodes and total nodes), and outcome features. The Kaplan–Meier method was used to evaluate the overall survival (OS) with the statistical software StatView v.4.55 (SAS Institute Inc., Cary, North Carolina, US). The OS was calculated from surgery time to death.

## 3. Results

A total of 27 patients with PM were managed at our institution over the four-year study period. Twenty-two patients met study inclusion criteria and underwent an ILND. The mean age of this cohort was 56 years (range 41–81 years), with a male-to-female ratio of 1 : 1.75.

### 3.1. Tumor

**Figure 1:**
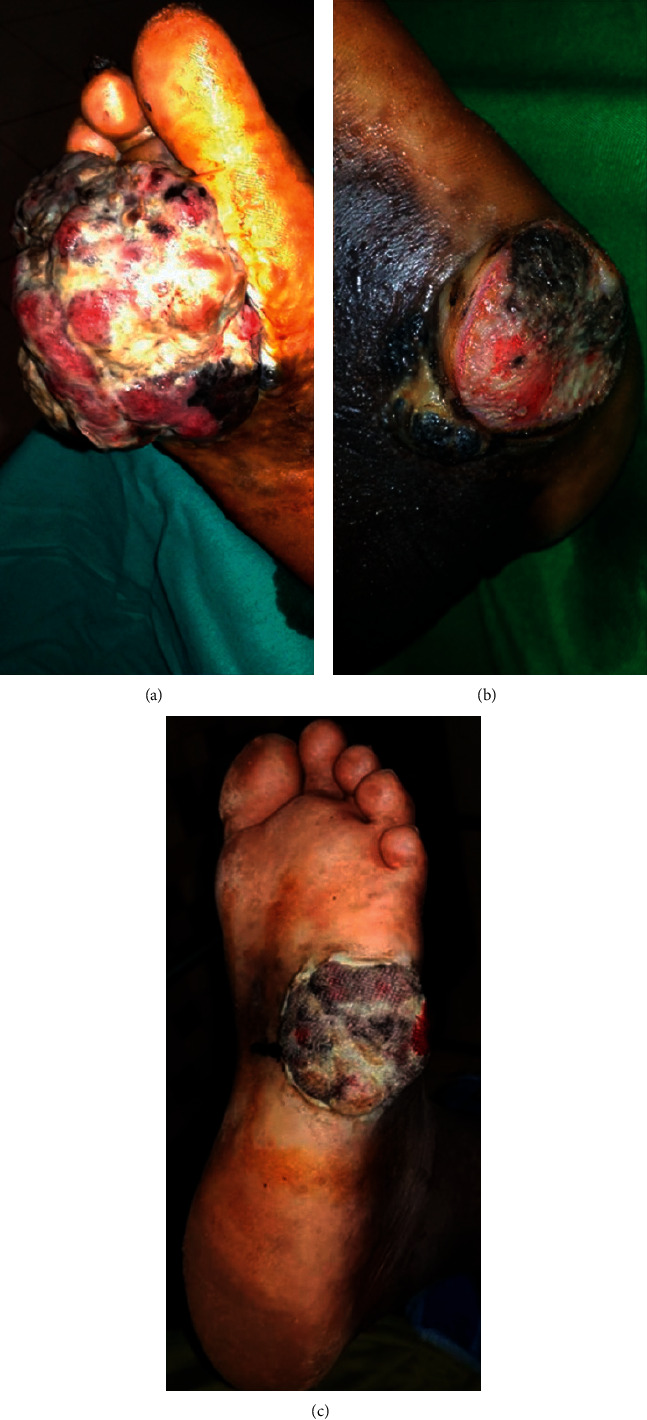
Ulceronodular plantar melanomas.

All 22 patients presented with large nodular primary tumors above four centimeters in height; six patients had tumor ulceration (Figure 1). Four patients presented with pulmonary metastases and one with cervicothoracic cutaneous metastasis. According to the AJCC 8^th^ edition classification [11], 18 patients with locoregional lymphadenopathy were stage III, and four patients with widely metastatic disease were stage IV.

### 3.2. Surgical Technique

**Figure 2:**
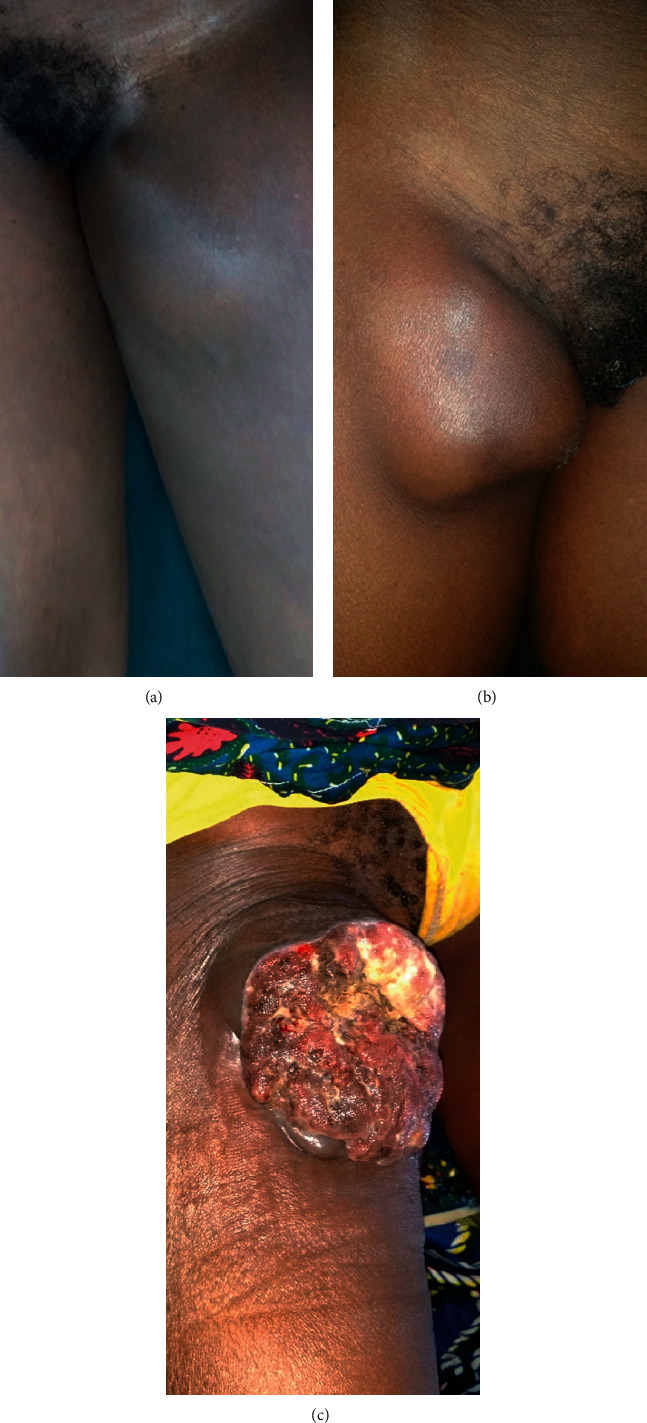
Inguinal metastasis lymphadenopathies aspects. (a) Palpable lymphadenopathy, (b) visible lymphadenopathy, and (c) ulcerous lymphadenopathy.

**Figure 3:**
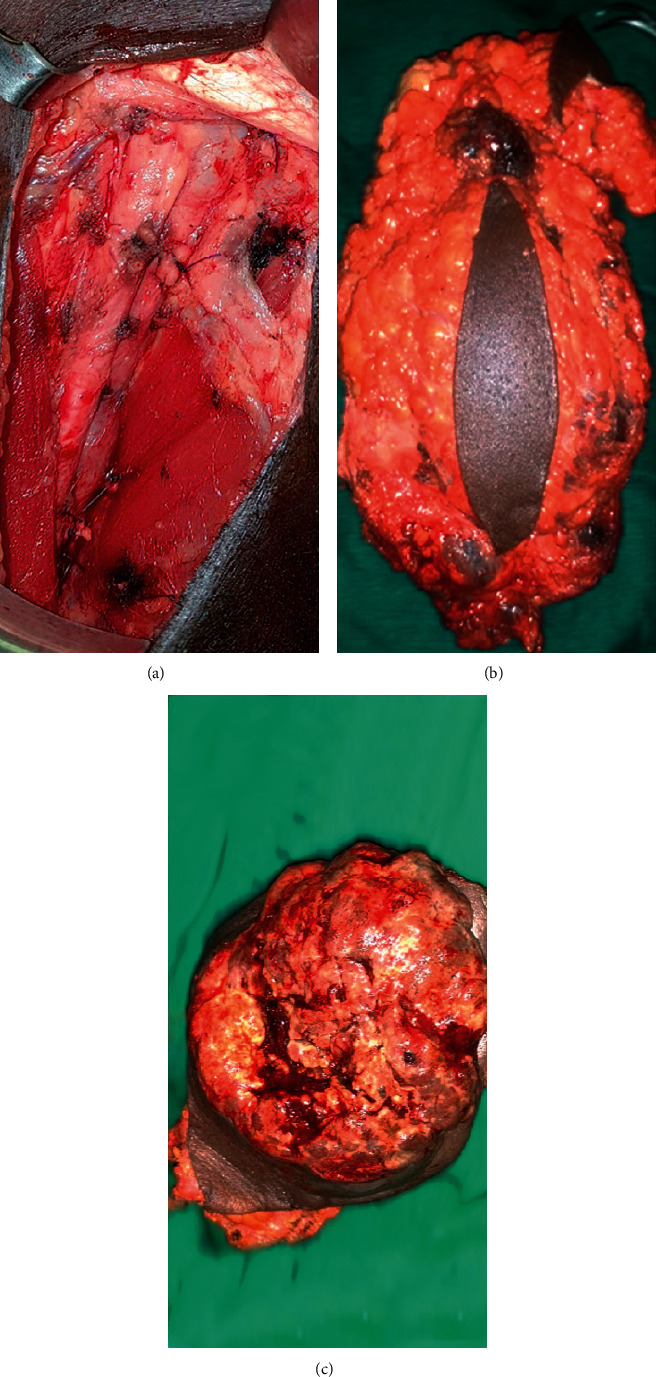
Ellipse-shaped inguinal lymph nodes dissection (ILND): (a) immediate postoperative aspect, (b) dissected lymph nodes and fat tissues, and (c) dissected ulceronecrotic lymph nodes.

ILND firstly and melanoma excision were done simultaneously. ILND was performed for 11 patients with visible lymphadenopathies, seven with palpable lymphadenopathies, and four with malignant ulcerous lymphadenopathies (Figure 2). We used a longitudinal S-shaped approach for palpable lymphadenopathy (*n* = 11). A large ellipse-shaped resection was used for visible and ulcerous lymphadenopathy (*n* = 11) for which an oval strip of skin with underlying lymph nodes was resected (Figure 3). A monobloc resection of superficial lymph nodes, the saphenous vein, and lymph nodes adjacent to the superficial femoral artery was performed. All operative wounds were closed over a vacuum suction drain.

### 3.3. Morbidity

No perioperative complications were reported. The large ellipse-shaped incision with tumor-free margins ensured a monobloc resection for large lymphadenopathy. Operative wounds healed in 46 days (21 to 90 days). In all cases, lymph leakage occurred for up to 10 days postoperatively. Eighteen local infections led to 16 wound dehiscences. The infections were probably due to *Staphylococcus aureus* since they healed well with antibiotics (covering Staphylococcus) and wound dressing.

### 3.4. Oncologic Pathology

Anatomopathological studies confirmed the diagnosis of melanoma in all cases. The tumor thickness was more than three millimeters in 17 cases, according to the Breslow index. The invasion was considered Clark IV in 20 cases. ILND resulted in excision of a mean of 12 lymph nodes (range: 8–34). All excised lymph nodes had metastatic disease.

### 3.5. Outcome

**Figure 4:**
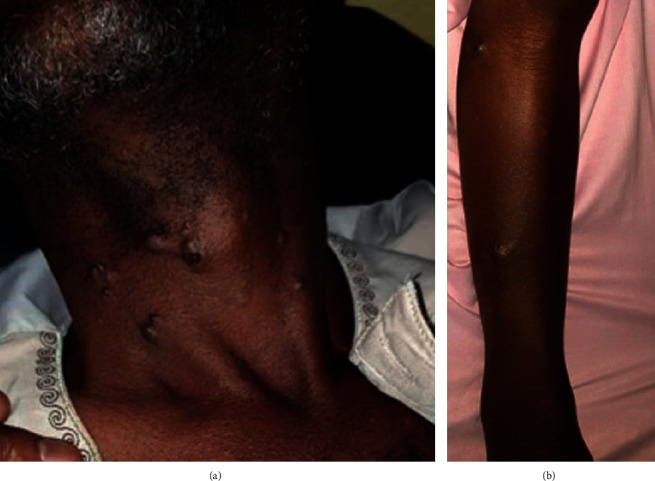
Cutaneous skip metastasis recurrences. (a) Cervical skip metastasis in a 57-year male and (b) leg skip metastasis in a 46-year female.

**Figure 5:**
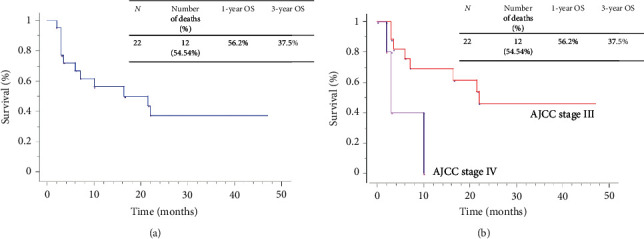
The overall survival (OS).

After 29 months of follow-up (range: 21 to 90 months), twelve patients had died, and seven were alive with cancer recurrence. Of the 12 patients who had died, one had cerebral and 11 had metastases. Five among 12 deaths were metastatic before surgery. Of the seven patients with cancer recurrence, two had palpable lymphadenopathy, two had skip metastases (Figure 4), and three had widespread metastases (Table 1). Three patients had complete remission without any clinical or radiological cancer recurrence. The median OS was 16.5 months for all 22 patients. OS rate for one and three-year were 56% and 38%, respectively. The AJCC stage IV median OS was three months. All stage IV patients died before one year. The median was better in AJCC stage III cases, where the median OS was 22 months (Figure 5).

## 4. Discussion

The current study has some limitations. As uncommon melanomas, our small sample size did not allow for adequate comparison between patients with stage III and stage IV disease. Limited availability of CT scan for the purposes of cancer staging may have underestimated the presence of metastatic disease in our cohort and lead to incorrect staging.

Few African studies emphasized the plantar site and the female predominance of melanomas in Africans [2, 3, 5]. This predominance is also found in African-Americans (AA) according to the US national databases [1, 12]. Compared to Caucasians, the disease is frequently diagnosed in later stages [1, 2, 12]. Diagnosis is often delayed due to inaccessibility of appropriate care [1, 3, 5]. In our country, poverty and beliefs in traditional bone-setters delay presentation to healthcare facilities [3, 9].

Misdiagnoses in smaller health centers and delayed specialized medical consultation also contribute to delayed presentation.

The current concept values the Morton sentinel node biopsy [13] and preoperative ultrasound examination [14]. Unnecessary lymphadenectomies have to be avoided [15]. The literature recommends lymphatic mapping and sentinel lymph node dissection (SLND) for early stages of melanomas [14, 15]. Our sample showed minimum AJCC stage III melanomas with large painful, ulcerous, and necrotic lymph nodes metastases. Without access to radiotherapy, surgery was the only option left to improve patients' quality of life and survival. In addition, our patients came from rural areas, without health insurance, and could not afford multiple surgeries. Thus, for large lesions, we do not recommend an initial core biopsy. For the primary tumor, we performed a large excisional biopsy with two centimeters margin and through the deep fascia according to the highest supposed Breslow and Clark grade. To perform ILND, multiple skin incisions techniques are described, including straight incisions [16, 17], ellipse-shaped incisions [16, 18–20], and two-separate incisions [21]. The straight incision seems to increase the risk of vascular injury and leg oedema [17, 18]. We believe that the ellipse-shaped incision and S-shaped incision provide better exposure to allow efficient lymph node dissection for large and ulcerous lymphadenopathy. When inguinal lymphadenopathies are large, visible with or without skin extension, the ellipse-shaped incision allows for effective cutaneous resection with tumor-free margins and lymph node dissection [16, 18]. The reported complications for inguinal lymph nodes dissection include pain, hematoma, local infection, local necrosis, and lymphoedema [7, 18, 20, 22]. Age above 55 years and body mass index above 25 are significantly associated with complications [20]. In terms of surgical complications, we observed persistent lymphorrhoea, which we believe was due to weak vacuum suction. Lymphorrhoea in our cohort led to a high rate of surgical site infection and wound dehiscence. No skin necrosis was observed.

The ILND in metastatic melanoma is controversial [7, 20, 23]. Hughes et al. [7] identified prognostic factors such as the number of involved superficial lymph nodes and the presence of extracapsular spread. Therefore, combined lymph nodes dissection reduces local recurrence but has not demonstrated a survival benefit [7, 20]. In our study, most patients presented with melanoma in its advanced stages, often with metastatic disease, and in some cases with malodorous, ulcerative, fungating lymphadenopathy. In the absence of radiotherapy in our country as an adjunctive or alternative treatment, we offered ILND to all patients with stage III and stage IV disease as a means of improving quality of life and improving overall survival.

The melanoma stage at presentation influences the prognosis [2, 24]. The 5-year survival rates for melanoma are better in Caucasians compared to Africans or AA [1]. The OS of our study was 56% in one year (Figure 4). It was higher in AJCC stage III (22 months) than AJCC stage IV (3 months). This was lower than reported by the SEER database, where 5-year OS for regional stage melanoma was 64.8% [8]. Even if the gain in OS is modest, the quality of life was greatly improved. Therefore, ILND for the AJCC stages III and IV remains an appropriate intervention in our practice.

## 5. Conclusion

The ILND procedure in advanced stages of plantar melanoma improved our patients' quality of life, notwithstanding short-term surgical complications. The development of adjuvant treatment in our facility might help to minimize recurrences and increase survival.

## Figures and Tables

**Table 1:** Recurrences after ILND.

Cases	Age	Sex	Recurrence (months)	Follow-up time (months)	Recurrence type and location
1	46	F	43	47	Leg skip nodules
2	50	M	30	46	Thigh lymphadenopathy (inguinal triangle vertex)
3	53	F	30	33.5	Pulmonary
4	45	M	25	26	Pulmonary
5	41	F	13	13	Inguinal lymphadenopathy
6	57	M	7.5	7.5	Face skin
					Pulmonary
7	56	F	1	3	Foot skin skip

## Data Availability

The data used during the current study are available from the corresponding author on reasonable request.

## Abbreviations

SEER: Surveillance, epidemiology, and end results
AJCC: Association Joint Committee on Cancer
NM: Nodular melanoma
SSM: Superficial spreading melanoma
LM: Lentigo maligna melanoma
ILND: Inguinal lymph nodes dissection
SLND: Sentinel lymph node dissection
PM: Plantar melanoma
AA: African-Americans
US: Ultrasonography
X-ray: Radiography
OS: Overall survival.
